# Neural Stem Cell Spreading on Lipid Based Artificial Cell Surfaces, Characterized by Combined X-ray and Neutron Reflectometry

**DOI:** 10.3390/ma3114994

**Published:** 2010-11-22

**Authors:** Martin Huth, Samira Hertrich, Gabor Mezo, Emilia Madarasz, Bert Nickel

**Affiliations:** 1 Ludwig-Maximilians-Universität, Department für Physik and CeNS, Geschwister-Scholl-Platz 1, 80539 Munich, Germany; E-Mails: m.huth@lmu.de (M.H.); Samira.Hertrich@physik.lmu.de (S.H.); 2 Research Group of Peptide Chemistry, Hungarian Academy of Science, Eötvös L. University, Pazmany P. stny. 1/A, 1117 Budapest, Hungary; E-Mail: gmezo@elte.hu (G.M.); 3 Laboratory of Cellular and Developmental Neurobiology, Institute of Experimental Medicine of Hungarian Academy of Science, Szigony u. 43, Budapest, H-1083, Hungary; E-Mail: madarasz.emilia@koki.hu (E.M.)

**Keywords:** supported lipid bilayers, biomimetics, bionanotechnology, functional coatings, self-assembly

## Abstract

We developed a bioadhesive coating based on a synthetic peptide-conjugate (AK-cyclo[RGDfC]) which contains multiples of the arginyl-glycyl-aspartic acid (RGD) amino acid sequence. Biotinylated AK-cyclo[RGDfC] is bound to a supported lipid bilayer via a streptavidin interlayer. Layering, hydration and packing of the coating is quantified by X-ray and neutron reflectometry experiments. AK-cyclo[RGDfC] binds to the streptavidin interlayer in a stretched-out on edge configuration. The highly packed configuration with only 12% water content maximizes the number of accessible adhesion sites. Enhanced cell spreading of neural stem cells was observed for AK-cyclo[RGDfC] functionalized bilayers. Due to the large variety of surfaces which can be coated by physisorption of lipid bilayers, this approach is of general interest for the fabrication of biocompatible surfaces.

## 1. Introduction

Control of cell attachment on surfaces is a fundamental requirement in biophysical situations where close contact between cells and a technical surface is required, such as in sensing applications or tissue engineering. The natural environment of a cell is composed of extracellular matrix (ECM) and the surfaces of other cells; therefore, an artificial surface coating has to mimic these conditions. Without proper attachment, cells sustain a special apoptotic fate called anoikis [1]. 

**Figure 1:**
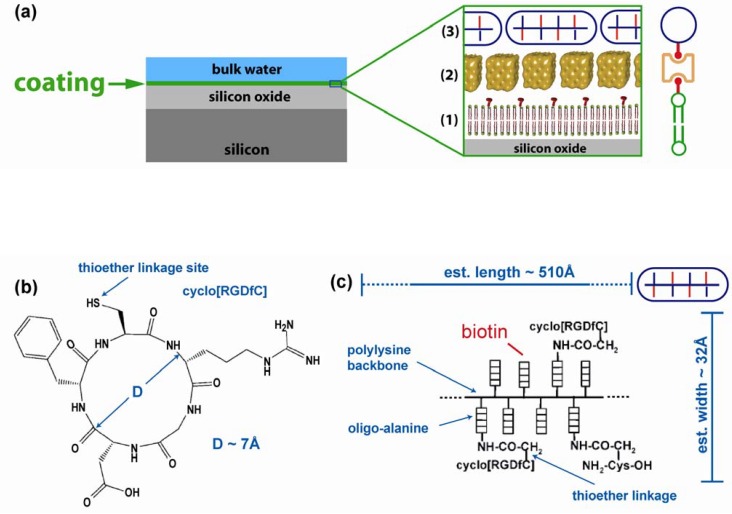
Schematics. (**a**) Sample structure. The lipid bilayer containing 2% of biotinylated lipids (1) is covered by a layer of streptavidin (2) which is bound to the lipid membrane by biotin anchors. A biotinylated adhesion peptide (AK-cyclo[RGDfC]) is bound on top of the streptavidin layer (3); (**b**) Chemical structure of the cyclic RGD-containing binding residue; (**c**) Architecture of the adhesion peptide. (b) and (c) are adapted from [2].

Here, cell attachment is promoted by a synthetic peptide-conjugate AK-cyclo[RGDfC]. AK-cyclo[RGDfC] is a novel, synthetic cell-adhesive peptide [2] comprising a poly-L-lysine backbone with oligo-D/L alanine side chains **(**AK, see Figure 1c) composed of both D- and L-enantiomers of alanine and carrying the adhesive end-motif cyclo[RGDfC] at the N-termini. As it was shown previously by CD analyses [3,4], the spacer built from raceme alanine residues results in non-structured peptide side-chains with increased sterical flexibility for the adhesive end-motif and improves the solubility of the carrier [5]. As a biologically active, cell adhesive moiety, the cyclo[RGDfC] cyclic pentapeptide (see Figure 1b) was chosen due to the strong affinity of cyclic RGD pentapeptides to selected types of cell surface integrins [6]. The Cys residue provided sites for conjugation and the introduction of a D-enantiomer phenyalanine into the peptide ring was thought to result in a rigid RGD motif [7] easily recognized by α_v_β_3_/α_v_β_5_/α_5_β_1_ integrins known to be present on the surface of a number of neural [8,9] and non-neural stem-like cells [2]. The resulting adhesive peptide conjugate was shown to support adhesion-based selection and serum-free propagation of neural stem cells[10]. This selectivity for adhering neural stem-like cells prompted us to functionalize lipid bilayers with AK-c(RGDfC) rather than peptides carrying linear RGD sequences [11] or laminin-motifs [12]. The cyclic RGD peptide was conjugated to a branched chain polypeptide AK through thioether linkage, which is an efficient tool for the preparation of polypeptide conjugates [13,14]. The RGD sequence is present in many extracellular matrix proteins (fibronectin, vitronectin, tenascin-C, *etc*.). It is responsible for the binding of ECM proteins to their receptors on cell surfaces. Previous studies of cells grown on AK-cyclo[RGDfC] adsorbed on SiO_2_ used biotinylated AK-cyclo[RGDfC] (see Figure 1c and Experimental), which is bound to streptavidin template on a biotinylated lipid bilayer (Figure 1a) [15]. Supported lipid bilayers (SLB) have proven to be an excellent experimental platform [16] to mimic functions of cell membranes. Since bare phospholipid bilayers are resistant to cell attachment in the first place, they are very well suited to study the amplification of cell attachment by functionalization with an adhesion promoter [12]. Using microscopy techniques, diffusion and protein binding can be studied [17]. For the characterization of the composition and layering of native or artificial membranes, a variety of surface techniques are available [18]. Structural properties can be accessed using reflection techniques [19,20,21]. Recently, X-ray experiments have revealed the structure of biotinylated phospholipid bilayers [15] decorated with streptavidin. This two-dimensional lipid-protein template is of special interest because of its biotin-binding properties, since biotin (vitamin B6, a cofactor of many enzymes) can be covalently bound to many proteins. The possibility to coat various surfaces with SLB [15,22,23,24,25,26] by physisorption, *i.e*., without the need for surface chemistry, is a strong point of this concept. Phospholipid membranes exist in different conformations, *i.e*., as monolayer, bilayer [22], and on some substrates as interdigitated bilayers [23]. Proteins can be embedded into the membrane or associated externally [15,27]. Note that SLBs can also be patterned by surface treatments [28,29,30], stamping [31] and optical post procession [32]. Dynamic patterning of SLB has been achieved using surface acoustic waves [33]. A sensor array coated with lipid bilayers was recently envisioned by Kumar *et al*. [34] and patterned attachment of human epithelial cells to SLB as a function of lipid composition was recently reported by Oliver *et al*. [35]. Here we study neural stem cell attaching to a synthetic cell surface based on a trilayer structure of adhesion protein, streptavidin interlayer and lipid bilayer. The nanostructure of this bioselective surface was investigated with X-ray and neutron diffraction experiments.

## 2. Results

### 2.1. Coating structure and arrangement

**Figure 2:**
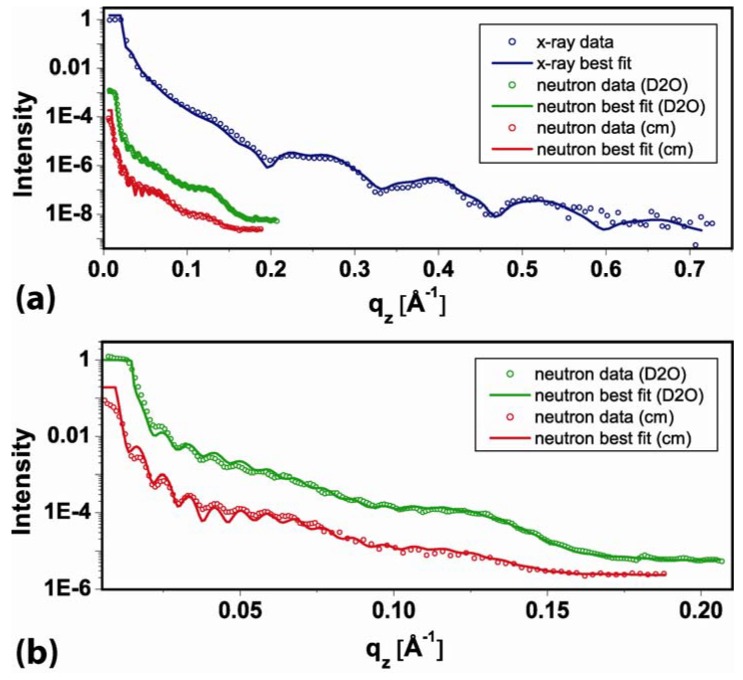
Reflectometry data and best fits of the SLB/streptavidin /AK-cyclo[RGD] trilayer in liquid environment (Figure 1a). (**a**) The reflected intensity is plotted against the momentum transfer q_z_. Intensity scale is logarithmic and normalized to a total reflection signal of 1. The reflectometry data are represented by open symbols, curves represent best fits. The neutron measurements are shown in green [*D_2_O*] and red [*cm*], the X-ray measurement is shown in blue; (**b**) Expanded view of the neutron data and best fits. Colors and symbols are as in (a).

The structural analysis combines X-ray and neutron reflectometry. The resolution σ[Å] in a reflectometry experiment can be estimated by π/q_z_ and is therefore limited by the maximum momentum transfer q_z_ that still yields a reflectometry signal. The momentum transfer q_z_ is given by q_z _= 4π/λ∙sin(2θ/2). Here, 2θ is the diffraction angle and λ is the wavelength. For X-ray, we achieve a q_z _= 0.7 Å^−1^ (Figure 2a), thus the internal structure of the layers can be determined with a resolution of σ = 5 Å. The intrinsic weak scattering contrast of proteins and water for X-rays can be improved using neutron beams [36]. For neutrons, the scattering length density (sld) of the solvent can be adjusted from −0.5∙10^−6^ Å^−2^ [100% H_2_O] [37] to 6.36∙10^−6^ Å^−2^ [100% D_2_O] [37] by using a mixture of water and heavy water. The sld of biomolecules are typically in between. The total sld of a layer is a combination of the sld of the molecules in the layer and the sld from hydration water. Thus, measuring in two different water mixtures allows separating the molecular density and layer hydration:

(1) $$sld[layer]=(1-h)\cdot sld[molecule]+h\cdot sld[D_{2}O/(H_{2}O+D_{2}O)]$$

Here *h* is the hydration of a layer in [%]. Therefore, in addition to the X-ray reflectometry experiment, we performed neutron reflectometry measurements in two different contrasts. One measurement was performed in 100% D_2_O [*D_2_O*], and one using a mixture which was contrast matched to the sld of SiO_2_ [57% D_2_O + 43% H_2_O, *cm*] (Figure 2). The value for the sld of the contrast matched medium was allowed to change between 3.3∙10^−6^ Å^−2^ and 4∙10^−6^ Å^−2^ to allow for small deviations in the mixing ratio in case that the previous medium might not have been exchanged completely. The fit yielded for the mixture a sld value of 3.9∙10^−6^ Å^−2^, indicating a 7.5% higher D_2_O fraction in the mixture. Combined fitting was performed using the Motofit [38] package in Igor®. A single set of structural parameters reproduces the neutron and X-ray reflectometry data. 

**Figure 3:**
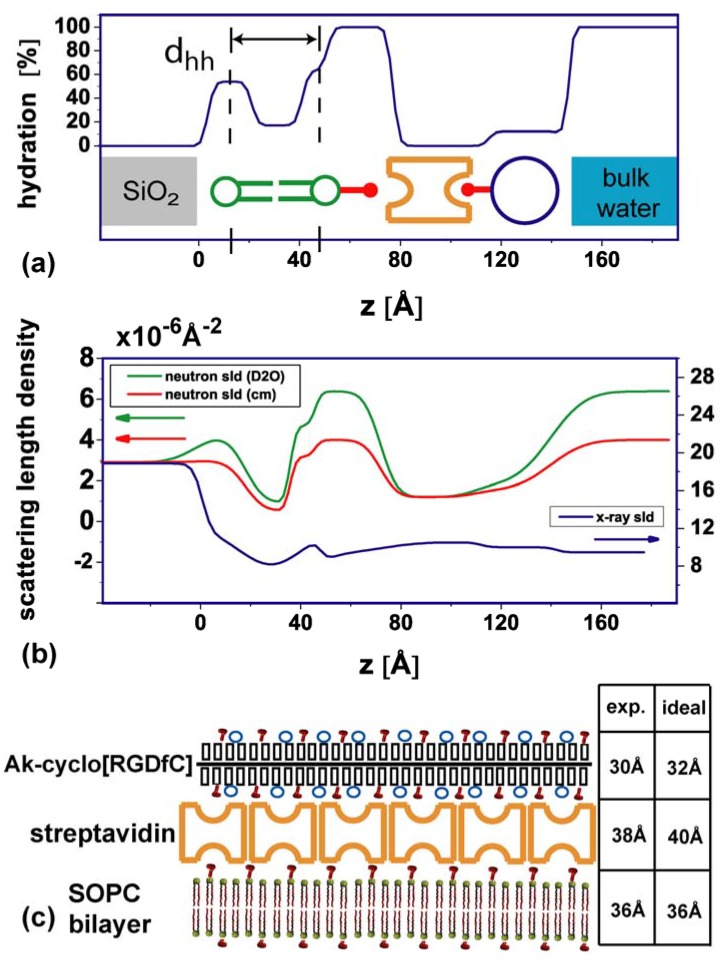
Scattering length density and hydration profile. (**a**) Hydration profile of the layers including a schematic of the layer components. d_hh_ indicates the head to head distance of the lipid bilayer; (**b**) Sld profiles of layers. Colored arrows indicate the respective y-axis; (**c**) Schematic of the synthetic peptide on top of the streptavidin interlayer. Red hooks depict the biotin residues, blue circles indicate the positions of a cyclo(RGDfC) residue. The bilayer-streptavidin interlayer is omitted. The table compares experimental findings and literature values.

In detail, first the thickness of the layers was determined from the X-ray data. The second step was to evaluate the hydration of the layers from the two neutron measurements. To estimate the hydration level, the molecular slds of lipids and streptavidin have been fixed according to the literature values [37,39] (Table 1). The neutron scattering length density of the AK-cyclo[RGDfC] was also fixed to the value of streptavidin. In an iterative process, the hydration values from the neutron measurements were used to refine the X-ray measurement. The X-ray sld of the AK-cyclo[RGDfC] was allowed to vary by 5% around the sld of streptavidin. The data (open circles) and final best fits (lines) are shown in (Figure 2). The scattering length density profiles are summarized in (Figure 3b). The hydration (Equation 1) of the layered system and a sketch of the constituents are illustrated in (Figure 3a). The parameters of the slab model are summarized in (Table 1).

**Table 1:** Parameters obtained from neutron and X-ray reflectometry data. The values describe the slab model of the sld and hydration profiles depicted in Figure 3.

	Neutron sld [10−6 Å−2] [f]	X-ray sld [10−6 Å−2] [f]	Thickness [Å]	hydration [%]
lipid heads	1.78	13.8	11	54 (lower) / 63 (upper)
lipid chains	−0.2	8	18	17
hydrated region	6.36 [a] / 3.9 [b]	9.45	26	100
streptavidin	1.2	10.5	38	0
AK-cyclo[RGDfC]	1.2	10.2 [b]	30	12

[a] D_2_O [b] fitted value, see text [f] fixed values

The head to head distance d_hh_ of the membrane of 36 Å (Figure 3a) is in agreement with values previously published [23]. A highly hydrated interlayer of 26 Å between the membrane and the streptavidin layer is observed. It contains the biotin anchor of the lipids (2% are biotinylated). Orthorhombic crystals of biotin yielded a unit cell with a long axis of 21 Å [40]. Structure determination of crystallized streptavidin-biotin complexes indicates that biotin can extend the dimension of streptavidin by 20% [41] which is in the order of 10 Å. The thickness of the close packed streptavidin layer (38 Å, no hydration) is in good agreement with the thickness of 40 Å found by Horton *et al*. [15].

For the AK-cyclo[RGDfC] layer, we obtain a thickness of 30 Å and a water content of 12%. In order to address the packing and configuration of the AK-cyclo[RGDfC] peptide on the streptavidin interlayer, we first estimated the length of the molecule in stretched conformation to 510 Å (Figure 1c) by multiplying the length of a lysine compartment (8.5 Å) [42] with the degree of polymerization DP_n_ =60 [2]. The width of the molecule is estimated to 32 Å by adding the length of two side chains, *i.e*., twice the sum of oligo-alanine (7 Å), the thioether linkage (2 Å) and the diameter of the cyclo[RGDfC] compound (7 Å). A schematic of the dimensions is given in Figure 1(c). From the comparison of the observed thickness of 30 Å with the estimated width of 32 Å, and the observation that the hydration of the layer is only 12%, we infer a configuration where the AK-cyclo[RGDfC] molecule is bound in an on edge configuration to the streptavidin surface. This implies an AK-cyclo[RGDfC] packing efficiency of 88%. Because of its length of 510 Å, the AK-cyclo[RGDfC] molecule in on edge configuration binds to several streptavidin molecules. A simplified 2D sketch of the arrangement is shown in Figure 3(c). 

### 2.2. Stem cell growth experiments

**Figure 4:**
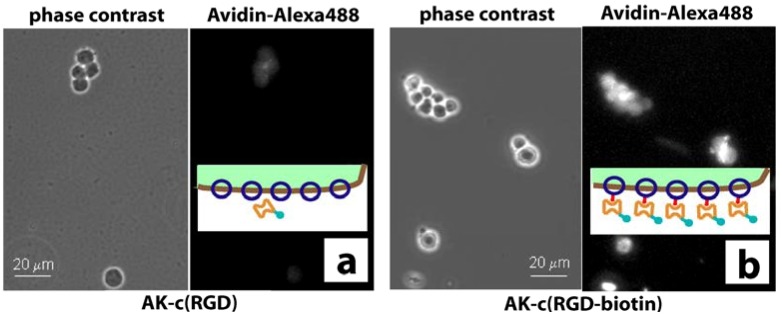
Phase-contrast and fluorescence microscopy (FM). (**a**) Neural stem cells are imaged by phase-contrast microscopy. Cells are first exposed to non-biotinylated AK-cyclo[RGDfC] peptides, and secondly to fluorescently labeled avidin, and finally imaged by FM. (**b**) Cell exposure to biotinylated AK-cyclo[RGDfC] enhances avidin absorption to cells. The insets illustrate the surface of a cell decorated with AK-cyclo[RGDfC] (shown in blue) or the biotinylated AK-cyclo[RGDfC] (shown in blue with red bars) and the binding to fluorescently labeled avidin (shown in orange, label shown in turquoise).

Neural stem cells carry a high density of integrins able to bind to the cyclo[RGDfC] domain of the synthetic adhesion molecule. In turn, the binding properties of the AK-cyclo[RGDfC] were evaluated by the binding of avidin to biotinylated and not-biotinylated AK-cyclo[RGDfC] (offered as cell surface receptors; Figure 4). Decoration of the stem cells with biotinylated or not-biotinylated AK-cyclo[RGDfC] was verified by subsequent binding of fluorescently labeled avidin to the cells after exposure to AK-cyclo[RGDfC]. In detail, the cells were suspended in artificial liquor solution (ACSF) and were incubated with AK-cyclo[RGDfC] (biotinylated or not-biotinylated) for 30 min at 37 °C, in suspension. After removing the excess peptides by three washes in ACSF, avidin-coupled fluorochrome (Alexa488-Avidin; Molecular Probes) was added to both preparations for 20 min. After rinsing, the binding of avidin to the biotinylated peptide was verified by fluorescence microscopic observations (Figure 4).

**Figure 5:**
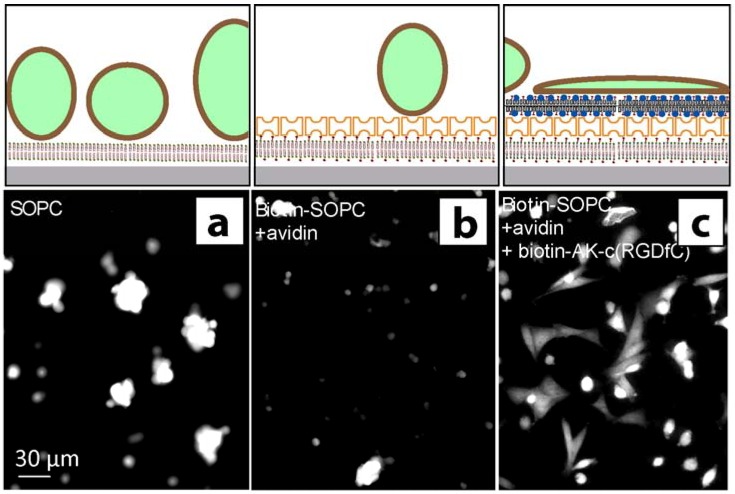
Fluorescence microscopy (FM) images of GFP expressing (fluorescent) radial glial cells. The FM images were recorded 4 h after cell seeding. (**a**) Radial glial cells attached to SOPC coated surfaces show a spheroid shape; (**b**) Cell attachment to an avidin functionalized biotinylated membrane is largely suppressed; (**c**) Rapid spreading of glial cells is observed for the biotin-AK-c[RGDfC] functionalized membrane. Schematics illustrate the conformation of the cells on the surface.

The spreading of green fluorescent protein (GFP) expressing neural stem cells upon exposure to different surface-coatings was investigated by fluorescence microscopy (Figure 5). For the plain SOPC membrane, a non-spreading, spheroid cell morphology and enhanced formation of cell aggregates indicated poor attachment of the cells to the surface (Figure 5a). Also, the lipid layers functionalized with avidin only did not support the attachment of neural stem cells (Figure 5b). Control experiments using non-biotinylated AK-cyclo[RGDfC], or biotinylated AK-cyclo[RGDfC] without an avidin interlayer, indicated some enhancement of cell attachment. Previous studies showed that AK-cyclo[RGDfC] adsorbs on SiO_2_ in loose-packed films [2], therefore we refer this enhancement to unspecific adsorption of AK-cyclo[RGDfC] to membrane defects or bare surface parts. For the biotinylated AK-cyclo[RGDfC], templated by an avidin-SLB support, improved and rapid spreading of the cells was observed (Figure 5c). The cell morphology indicates cell attachment to the RGD motives.

## 3. Discussion

Combining neutron and x-ray reflectometry allowed us to resolve the trilayer structure of the SLB/streptavidin/AK-cyclo[RGDfC] system with a resolution of *ca.* 5 Å. The thickness of the individual layers is within the range expected from the molecular dimensions of the respective layers. In this sense, Figure 3c is a structure model rather than a cartoon. The analysis of the hydration level of the individual layers is a strong point of this approach. The hydration of the lipid bilayer chain part (17%) indicates that the bilayers contain some defects, which may be the origin of the stem cells attaching, but not spreading, to the SLB, cf. Figure 5a. Apparently, the subsequent avidin functionalization closes these defects, since attachment to this layer is largely reduced compared to the bare SLB. Here, the reflectometry measurements indicate a rather tight and homogeneous layer without water, in line with the observation that under these conditions a dense crystalline layer forms [15]. In this context, one should note that contrast variation allows only to measure excess water which can be exchanged by changing the buffer. Furthermore, the water in contact with the lipid headgroups has been separated from the streptavidin layer. 

The most interesting finding is the low hydration (12%) and close packing of the cyclo-RGD containing layer, which matches typical values for lipid bilayer chains determined by neutron reflectometry. This value suggests that the hydrophobic character of the peptide backbone and side chains, in combination with the specific binding to streptavidin and the hydrophilic binding motif exposed to the buffer solution, results in a dense and oriented packing, (see Figure 5c). This could be an advantage compared to e.g., direct binding of the RGD motif to a hydrophilic high energy surface such as SiO_2_. In agreement with high structural homogeneity, we have observed cells spreading on this surface, indicating a proper attachment to the exposed RGD motifs. 

## 4. Experimental Section

Commercially available silicon wafers with a thermally grown oxide layer of 100 nm thickness, purchased from SiMat, Germany, were used as substrates for the X-ray reflectometry and the cell adhesion experiments. The neutron reflectometry measurement was performed using a polished silicon block (10 × 5 × 1 cm) with a 60 nm thermally grown oxide layer. Substrates were cleaned by standard solvents and wet chemistry steps. In detail, wafer pieces were immersed in acetone and isopropanol, followed by sonication in deionized water (DI), followed by an alkaline step (NH_4_OH:H_2_O_2_:DI mixed 1:1:5 at 80 °C, 20 min), an acidic step (HCl:H_2_O_2_:DI mixed 1:1:5 at 80 °C, 15 min), and again the alkaline step. Lipid solutions were produced by mixing SOPC (1-stearoyl-2-oleoyl-sn-glycero-3-phosphocholine) lipids with 0.5 mol% of DHPE (1,2-dihexadecanoyl-sn-glycero-3-phosphoethanolamine) lipids labeled with a fluorescent dye (Oregon Green) and alternatively adding 2 mol% biotin-X-DHPE (N-((6-(biotinoyl)amino)hexanoyl)-DHPE, triethylammonium salt). The lipids were mixed in chloroform and dried with nitrogen. The supported lipid bilayers were produced by spincoating. The appropriate lipid solution was dissolved in isopropanol (1.5 mg/mL) and spincoated on the bare substrate with a BLE delta spincoater, using maximum acceleration and a ramp with 3 sec at 2,000 rpm followed by 60 sec at 3,000 rpm. To minimize solvent residues, the samples were kept in vacuum at room temperature for at least 4 h. Sterile tissue culture plates were used for transport and storage of the samples to prevent bacterial contamination. Mounting of the samples in the microfluidic chamber [43], as well as the change of buffer-solutions or DI water was done in a sterile environment. To form the supported lipid bilayer, the fluidic chamber embedding the lipid coated wafer was filled with deionized water and kept in the dark overnight at ambient temperature. After extensive rinsing, excess lipids in solution and multilayers forming on the surface were removed, using fluorescence microscopy to control. The DI water was first replaced by PBS buffer (pH = 7.4). Then 200 µL of streptavidin (Sigma-Aldrich) dissolved in PBS buffer (40 µg/ml) was injected into the fluidic chamber, which was stored in the dark overnight at ambient temperature. Finally, the Streptavidin solution was replaced by DI water and extensively rinsed. The AK-cyclo[RGDfC] peptide-conjugate was synthesized according to [2] (Research Group of Peptide Chemistry, HAS, Budapest) using the following protocol (see [2,13,14] for details). The peptides (1 µg/µL) were biotinylated by overnight incubation with SNHS-biotin (2 µg/µL; Sigma) in 1 M phosphate buffer (pH = 7.6). The excess biotin and salts were removed by 24-hour dialyzation (3500 kDa dialyzing membrane; Serva) against distilled water with three fluid changes. After dialyzation, the protein content was determined and the preparation was occasionally concentrated by vacuum dialyzation. Biotinylated AK-cyclo[RGDfC] molecules (10 µg/mL in DI water) were injected into the microfluidic chamber and were let to bind overnight in the dark. After incubation, the chamber was rinsed with DI water to remove excess AK-cyclo[RGDfC] molecules, which were not bound to the streptavidin layer. Samples were freshly prepared for each experiment.

The X-ray scattering experiments have been carried out at the Hamburger Synchrotronstrahlungslabor (Hasylab) in Hamburg, Germany (beamline D4). The wavelength was λ = 0.62 Å and measurements on samples representing subsequent steps of the sample preparation series were carried out at different positions of the sample surface to avoid beam damage effects. Neutron experiments were performed at the NREX experiment at the FRM II in Garching, Munich, Germany. The neutron wavelength used was λ = 4.26 Å.

GFP-expressing sub-clone of NE-4C embryonic neuroectodermal stem cells [44] (GFP-4C; ATTC CRL-2926) were propagated on poly-L-lysine coated culture dishes in Minimum Essential Medium (MEM, Sigma, Hungary) supplemented with 5% fetal calf serum, while radial glia-like (RG-1) cells cloned from GFP-expressing fetal mouse forebrain [10] were cultivated on AK-c(RGDfC)-coated dishes in DMEM/F12 (1:1; Sigma Hungary) supplemented with B-27 (Invitrogen, Hungary) and 10 ng/ml EGF. After harvesting by trypsinization, the cells were suspended in artificial cerebrospinal fluid (ACSF). After 20 min recovery at 37 °C in a gas atmosphere containing 5% CO_2_, the cells (at a density of 10^5^ cells/mL) were introduced into lipid-coated chambers or were treated with plain or biotinylated AK-cyclo[RGDfC] (0.25 µg/mL) for 30 min, in suspension at mild agitation in a CO_2_-incubator.[44]

## 4. Conclusions

The combination of x-ray and neutron reflectometry experiments allows quantifying the layered structure of the AK-cyclo[RGDfC]/streptavidin/SLB system in terms of layer thickness, hydration, and packing. AK-cyclo[RGDfC] binds to the streptavidin template in a stretched on edge orientation. The high molecular packing efficiency of 88% provides a dense layer containing the RGD adhesion motive. Neural stem cells readily spread on such surfaces, while the bare SLB does not support the attachment of neural stem-like cells. A large variety of surfaces can be coated by lipid bilayers using physisorption and various techniques for bilayer patterning are available. Using this approach, the preparation of highly effective coatings for stem cells growth on various chemically inert surfaces is possible.
